# Causal mechanisms and balancing selection inferred from genetic associations with polycystic ovary syndrome

**DOI:** 10.1038/ncomms9464

**Published:** 2015-09-29

**Authors:** Felix R. Day, David A. Hinds, Joyce Y. Tung, Lisette Stolk, Unnur Styrkarsdottir, Richa Saxena, Andrew Bjonnes, Linda Broer, David B. Dunger, Bjarni V. Halldorsson, Debbie A. Lawlor, Guillaume Laval, Iain Mathieson, Wendy L. McCardle, Yvonne Louwers, Cindy Meun, Susan Ring, Robert A. Scott, Patrick Sulem, André G. Uitterlinden, Nicholas J. Wareham, Unnur Thorsteinsdottir, Corrine Welt, Kari Stefansson, Joop S. E. Laven, Ken K. Ong, John R. B. Perry

**Affiliations:** 1 MRC Epidemiology Unit, University of Cambridge School of Clinical Medicine, Box 285 Institute of Metabolic Science, Addenbrooke's Hospital, Cambridge Biomedical Campus, Cambridge CB2 0QQ, UK; 2 23andMe Inc., Mountain View, California 94043, USA; 3 Department of Internal Medicine, Erasmus MC, Rotterdam 3015 GE, The Netherlands; 4 deCODE Genetics/Amgen, Sturlugata 8, IS-101 Reykjavik, Iceland; 5 Department of Anaesthesia and Center for Human Genetic Research, Massachusetts General Hospital, Boston, Massachusetts 02114, USA; 6 Department of Paediatrics, University of Cambridge School of Clinical Medicine, Box 181, Cambridge Biomedical Campus, Cambridge CB2 0QQ, UK; 7 Institute of Biomedical and Neural Engineering, School of Science and Engineering, Reykjavík University, Menntavegur 1, 101 Reykjavík, Iceland; 8 MRC Integrative Epidemiology Unit at the University of Bristol, Bristol BS8 2BN, UK; 9 School of Social and Community Medicine, University of Bristol, Oakfield House, Bristol BS8 2BN, UK; 10 Human Evolutionary Genetics, CNRS URA3012 Institut Pasteur, 28 rue du Dr. Roux, 75724 Paris Cedex 15, France; 11 Department of Genetics, Harvard Medical School, Boston, Massachusetts 02115, USA; 12 Division of Reproductive Medicine, Department of Obstetrics and Gynaecology, Erasmus MC, Rotterdam, 3015 GE, The Netherlands; 13 Faculty of Medicine, University of Iceland, IS-101 Reykjavik, Iceland; 14 Division of Endocrinology, Metabolism and Diabetes, University of Utah School of Medicine, Salt Lake City, Utah 84112, USA

## Abstract

Polycystic ovary syndrome (PCOS) is the most common reproductive disorder in women, yet there is little consensus regarding its aetiology. Here we perform a genome-wide association study of PCOS in up to 5,184 self-reported cases of White European ancestry and 82,759 controls, with follow-up in a further ∼2,000 clinically validated cases and ∼100,000 controls. We identify six signals for PCOS at genome-wide statistical significance (*P*<5 × 10^−8^), in/near genes *ERBB4/HER4*, *YAP1*, *THADA*, *FSHB*, *RAD50* and *KRR1.* Variants in/near three of the four epidermal growth factor receptor genes (*ERBB2/HER2*, *ERBB3/HER3* and *ERBB4/HER4)* are associated with PCOS at or near genome-wide significance. Mendelian randomization analyses indicate causal roles in PCOS aetiology for higher BMI (*P*=2.5 × 10^−9^), higher insulin resistance (*P*=6 × 10^−4^) and lower serum sex hormone binding globulin concentrations (*P*=5 × 10^−4^). Furthermore, genetic susceptibility to later menopause is associated with higher PCOS risk (*P*=1.6 × 10^−8^) and PCOS-susceptibility alleles are associated with higher serum anti-Müllerian hormone concentrations in girls (*P*=8.9 × 10^−5^). This large-scale study implicates an aetiological role of the epidermal growth factor receptors, infers causal mechanisms relevant to clinical management and prevention, and suggests balancing selection mechanisms involved in PCOS risk.

## Abstract (web summary)

This paper describes the largest genome-wide association study to date on polycystic ovary syndrome (PCOS), a common reproductive disorder in women. Six genetic loci—including known targets of cancer chemotherapy—were identified, and the authors infer causal and balancing selection mechanisms involved in PCOS risk and susceptibility.

## Graphical abstract

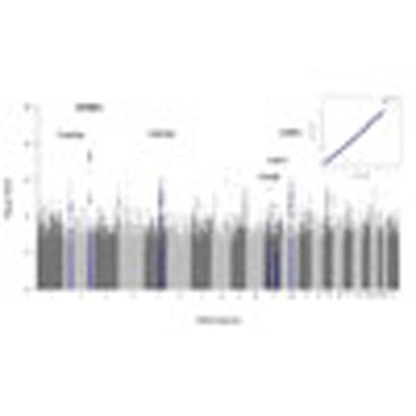

Polycystic ovary syndrome (PCOS) is a common reproductive disorder in women that is defined by two out of three criteria: (1) menstrual irregularity (oligo-ovulation or anovulation), (2) hyperandrogenism (clinical or biochemical) and (3) polycystic ovarian morphology^1^,^2^. Phenotypic heterogeneity between cases has limited the ability to make definitive conclusions regarding its aetiology and pathophysiology. Obesity is associated with PCOS, but its causal role has yet to be determined^3^; alternative explanations include reverse causality (that is, PCOS increases susceptibility to weight gain) and synergistic but independent roles for obesity and PCOS in infertility^4^. Hence, the role of lifestyle modification to prevent or reverse the reproductive abnormalities of PCOS is not well established^5^,^6^. Furthermore, although there is extensive evidence linking insulin resistance to PCOS, it is widely considered that the cellular and molecular mechanisms of insulin resistance in PCOS differ from those in other common insulin-resistant states such as obesity and diabetes^3^,^7^. Consequently, the role of insulin sensitisation therapy in PCOS remains limited to the prevention of cardiovascular disease and type 2 diabetes (T2D)^8^,^9^.

Genetic studies could identify underlying genes and pathways, and thereby provide insights into the aetiology of PCOS. The results of candidate gene studies have been inconclusive, in large part due to underpowered studies, lack of replication and limited prior understanding of its pathogenesis^10^. Two, large genome-wide association studies (GWAS) for PCOS in overlapping Han Chinese populations identified in total 11 genomic loci^11^,^12^. Although these loci were enriched for candidate genes related to insulin signalling, steroid hormone regulation and T2D, and also for genes related to calcium signalling and endocytosis, the ability to make mechanistic interpretations from those findings was limited and only a few of these loci have been replicated in PCOS cases of European ancestry^13^,^14^,^15^,^16^,^17^. Furthermore, the striking paradox of a highly heritable yet common condition that impairs fertility has led to multiple theories for a balancing advantage of PCOS susceptibility^4^. Suggested mechanisms include enhanced fetal growth and development^18^ or reproductive advantages, such as earlier pubertal maturation^19^ or retarded ovarian ageing leading to a sustained reproductive lifespan^20^.

Here we present a large-scale GWAS for PCOS in cases and controls of Caucasian European ancestry. As well as being the largest such study to date, we use dense imputation of genotypes to better implicate the probable genes underlying the association signals. As the GWAS is based on self-reported PCOS cases, we present follow-up in additional studies of clinically validated cases. We find six genetic loci associated with PCOS, highlighting aetiological roles for the epidermal growth factor receptors (EGFRs) and for the pituitary-derived gonadotrophins. Furthermore, using a genetic instrumental variable approach (i.e., Mendelian randomization)^21^, we infer causal roles in PCOS aetiology for higher body mass index (BMI), higher insulin resistance and lower serum sex hormone binding globulin (SHBG) concentrations. Finally, we find a robust association between menopause age-delaying alleles and higher risk of PCOS, suggesting a potential evolutionary advantage for PCOS genetic susceptibility.

## Results

### Genome-wide association signals for PCOS

**Figure 1:**
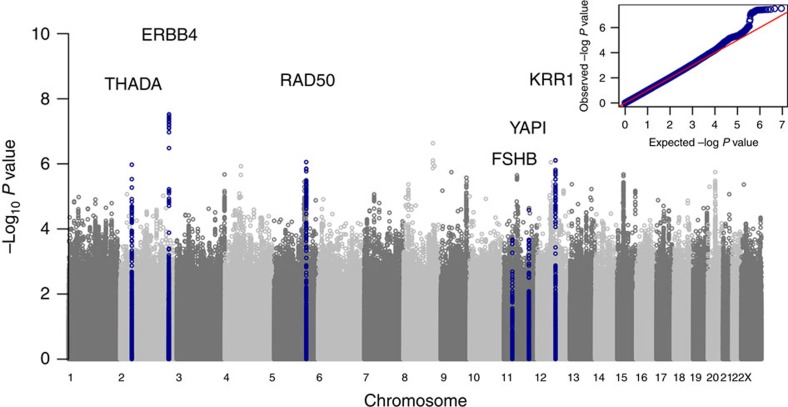
Manhattan and QQ plots displaying PCOS genome-wide association results. Results shown are from discovery phase only.

Six independent common signals reach genome-wide significance (logistic regression *P*<1 × 10^−8^) for association with PCOS in the meta-analysis of discovery and follow-up studies (Table 1, Fig. 1 and Supplementary Fig. 1); four are novel signals and two represent refinements of previously reported signals at the *YAP1* and *THADA* loci. All signals show at least nominally significant (*P*<0.05) directionally concordant associations in the follow-up studies of clinically validated PCOS cases, with no significant heterogeneity by PCOS case definition (Supplementary Table 2).

Our strongest novel PCOS signal (rs1351592, odds ratio: 1.18 (1.13–1.23), *P*=1.2 × 10^−12^) is intronic in *ERBB4/HER4*, which encodes a member of the EGFR family. Notably, we find further sub-genome-wide significant signals in/near genes encoding two of the other three EGFR family members: rs7312770 (*P*=2.1 × 10^−7^) in/near *ERBB3/HER3* is correlated (*r*^2^=0.40) with the reported PCOS signal (rs705702) at 12q13.2 and rs7218361 (*P*=9.6 × 10^−7^) is a low-frequency variant ∼200 kb downstream of *ERRB2/HER2*.

Our second strongest novel signal (rs11031006, *P*=1.3 × 10^−9^) lies near *FSHB*, which encodes the hormone-specific β-subunit of follicle stimulating hormone (FSH), a key promoter of ovarian follicle growth and oestrogen production. Interestingly, in deCODE samples, the PCOS-susceptibility allele at rs11031006 is also robustly associated with lower circulating FSH concentrations (*β*=−0.089 s.d. per allele, *P*=9.2 × 10^−10^, *n*=15,586 women), higher luteinizing hormone (LH) concentrations (*β*=0.115 s.d. per allele, *P*=3.6 × 10^−15^, *n*=17,469 women) and higher LH/FSH ratio (*β*=0.272 s.d. per allele, *P*=5.94 × 10^−68^, *n*=14,310 women). This variant represents the strongest association signal for FSH, LH and LH/FSH ratio at this *FSHB* locus. Notably, a variant rs12294144 correlated with the PCOS risk allele is reportedly associated with later age at menopause^22^. Furthermore, FSH signalling was implicated in PCOS in the Han Chinese GWAS study through association with the FSH receptor gene *FSHR*^12^. However, that signal is only weakly associated with PCOS in our data (Table 2, rs2268361, *P*=1.6 × 10^−2^).

Our third novel signal (rs13164856, *P*=3.5 × 10^−9^) is near *RAD50*, which encodes a protein involved in DNA double-strand break repair. Fourth, rs1275468 (*P*=1.9 × 10^−8^) indicates a novel PCOS signal near *KRR1*, which encodes a ribosome assembly factor.

### Previously reported PCOS loci

Of the 11 PCOS signals reported in Han Chinese^11^,^12^, we observe directionally consistent associations for 10 variants, 6 of which are at least nominally associated (*P*<0.05) in our discovery GWAS samples (Table 2). Effect estimates are consistently smaller in our data, and in several instances the risk allele frequency is markedly different between these Han Chinese and white European populations. At three reported Han Chinese PCOS loci (*YAP1*, *THADA* and *DENND1A*), we observe different lead signals in our white European samples (Table 1). Our lead *YAP1* signal, rs11225154 intronic to *YAP1*, is highly correlated with the reported *YAP1* signal (*r*^2^=0.74 with rs1894116) and reaches genome-wide significance in our combined discovery and follow-up analysis (*P*=7.6 × 10^−11^). Our lead *THADA* signal, rs7563201 intronic to *THADA*, also reaches genome-wide significance (*P*=3.3 × 10^−10^) but is only weakly correlated with the reported *THADA* signal (*r*^2^=0.08 with rs13429458). Our lead *DENND1A* signal (rs10760321) is also weakly correlated with the reported *DENND1A* signal (*r*^2^=0.02 with rs2479106) but was not confirmed in our follow-up samples. These findings probably reflect differences in allelic structure between Chinese and European ancestry groups, as has been concluded by other investigators^15^, and limit the potential for conventional meta-analysis across these populations.

### Mendelian randomization analyses

**Figure 2:**
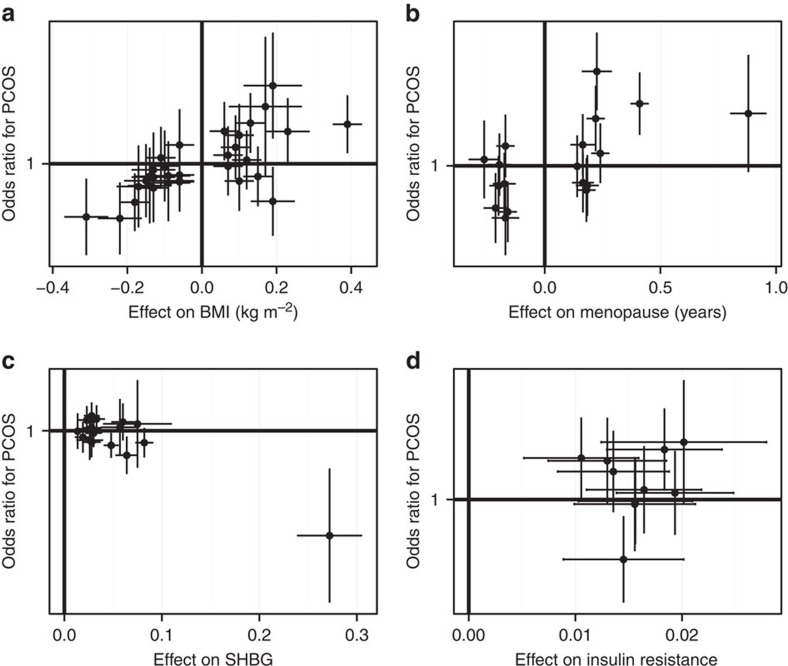
Scatter plots of the associations between four significant intermediate traits. Panels show (**a**) BMI, (**b**) age at menopause, (**c**) SHBG and (**d**) insulin resistance, in each case showing the associations between the SNP and the trait of interest, and the odds ratio for PCOS for that SNP, with the attendant 95% confidence intervals.

Our Mendelian randomization analyses indicate causal effects on PCOS aetiology for higher BMI (odds ratios: 1.90 per +1 s.d., 95% confidence interval: 1.55–2.34, *P*=2.5 × 10^−9^), higher insulin resistance (1.11 per +1 s.d., 1.05–1.19, *P*=6 × 10^−4^) and lower circulating SHBG concentrations (0.86 per +1 s.d., 0.78–0.93, *P*=5 × 10^−4^) (Table 3). Furthermore, the multiple allele score for menopausal age is positively associated with PCOS risk (1.60 per +1 s.d., 1.35–1.91, *P*=1.6 × 10^−8^), indicating a common biological mechanism that promotes both PCOS susceptibility and later menopause. Our sensitivity analyses show apparent dose–response effects across individual single-nucleotide polymorphisms (SNPs) in each of these scores (Fig. 2) and Funnel plots show no SNPs with outlier effects (Supplementary Fig. 3). In contrast, we find no evidence for causal effects on PCOS for birth weight (*P*=0.22) or age at menarche (*P*=0.23).

### Other biological mechanisms associated with PCOS

By systematic testing of all GWAS SNPs across all known biological pathways using meta-analysis gene-set enrichment of variant associations (MAGENTA) software, we find one further pathway (ATP-binding cassette transporters) that is enriched for PCOS-associated variants. This pathway includes the genome-wide significant signal at the DNA repair gene *RAD50* (rs13164856) and 37 other genes.

The PCOS-susceptibility alleles at our six PCOS loci are also consistently associated with higher anti-Mullerian hormone (AMH) concentrations in girls (cumulative score: *P*=8.9 × 10^−5^) (Supplementary Fig. 3). However, none of these six genome-wide significant PCOS loci (nor any of the four suggestive loci) overlap with reported signals of positive selection and we can find no evidence of polygenic selection on the set of six loci considered together (*P*=0.22) (Supplementary Note). Furthermore, these PCOS SNPs (or their proxies) are not associated with BMI (in aggregate: *P*=0.22).

## Discussion

This large-scale genetic study reveals a number of insights into the aetiology and pathophysiology of PCOS. The findings from our Mendelian randomization analyses have perhaps most immediate relevance for treatment and prevention^21^, as these infer causal roles of greater BMI and insulin resistance. The role of interventions aimed at these targets in PCOS is debated. A recent US Endocrine Society Task Force found evidence that lifestyle modification reduces fasting blood glucose and insulin concentrations in women with PCOS but has uncertain effects on the key clinical features of PCOS, including reproductive outcomes^5^. The same conclusion was reached for the use of the insulin sensitizer Metformin in PCOS^5^,^23^. Conversely, a recent non-quantitative synthesis of dietary interventions positively concluded that weight-reducing diets have clinical benefits in PCOS^24^. The limitations of Mendelian randomization analyses are well-recognized; its major assumptions regarding lack of heterogeneity and pleiotropy are supported by the consistency of our findings across individual SNPs. Furthermore, the reported inverse association between the insulin resistance genetic score and BMI^25^ might attenuate our observed positive univariate effects of these traits on PCOS risk. Other uncertainties remain, such as possible canalization and age-specific effects. Our findings should encourage the development and testing of more effective interventions to lower BMI and insulin resistance in women with PCOS.

Our findings also infer a causal protective role of SHBG for PCOS, as has been reported for T2D^26^. SHBG regulates the bioavailability of testosterone. Therefore, genetic variants that lower circulating SHBG concentrations might directly modify the key hyperandrogenic phenotype of PCOS and also the related adverse metabolic profile^27^. Circulating SHBG concentrations rise markedly with the introduction of combined oral contraceptive pills, which are used by many women with PCOS for treatment of menstrual irregularity, acne and hirsutism^28^; however, there are as yet no therapeutic agents that specifically target SHBG concentrations or activity. Despite the lack of any overlap between SNPs used in the SHBG and insulin resistance scores, it remains possible that these traits might lie on the same causal pathway, in which case joint interventions might have synergistic effects.

Our novel genetic signals indicate a major role of the EGFRs in the pathogenesis of PCOS. There are four members of the EGFR family: EGFR, ERBB2, ERBB3 and ERBB4 (the last three are also known as the human epidermal receptors: HER-2, HER-3 and HER-4)^29^. These receptors form ligand-activated homo- or heterodimers with each other, which activates tyrosine kinase, and in cancer cells result in cell proliferation, blocking of apoptosis, activation of invasion and metastasis, and stimulation of neovascularization. EGFR signalling mediates LH-induced steroidogenesis, which in turn promotes late follicular maturation^30^,^31^. EGFRs are overexpressed in ovarian cancer^32^,^33^ and repression of ERBB2/HER-2 determines the breast cancer response to the oestrogen receptor inhibitor tamoxifen^34^. Small molecules or monoclonal antibodies that block EGFR activation are effective cancer chemotherapy agents^29^. Variable reported associations between PCOS and risks of breast, endometrial and ovarian cancers are limited by small sample sizes and confounding due to related risk factors such as nulliparity, infertility and its treatment, anovulation and obesity^3^. Our findings provide a possible genetic link between PCOS and cancer risk, and also suggest potential ovary-targeted pharmaceutical interventions for treatment of PCOS.

The novel PCOS locus at *FSHB* represents striking biological complementarity to the locus at the FSH receptor gene *FSHR* reported in Han Chinese^12^. However, the impact of that *FSHR* variant on FSH receptor activity is unclear and that locus shows only nominal association in our data, likely to be due to population differences in genetic architecture. Non-synonymous variants in *FSHR* that confer lower FSH receptor activity are inconsistently associated with PCOS^35^. We show that the PCOS-susceptibility allele at *FSHB* is robustly associated with a higher LH/FSH ratio, which is the hallmark biochemical PCOS trait that promotes ovarian androgen production and arrests follicular growth^36^. Although the high LH/FSH ratio observed in PCOS might be exacerbated by central feedback effects of peripheral hyperandrogenemia^37^, our findings establish a co-primary neuroendocrine pathogenesis of PCOS.

Our findings inform the long-standing debate regarding the evolutionary paradox of PCOS as a common yet highly heritable disorder characterized by infertility. We cannot find evidence for recent, strong positive selection of PCOS-susceptibility alleles; however, available tests may be insensitive to detect signals that affect complex traits^38^,^39^. The robust association between menopause age-raising alleles and PCOS susceptibility implicates a common mechanism that retards ovarian ageing. GWAS studies for age at menopause has highlighted a key role for DNA repair pathways^22^,^40^ and their putative relevance to PCOS is supported by the novel PCOS locus near to *RAD50*, a gene that is involved in DNA double-strand break repair and is mutated in the Nijmegen breakage syndrome-like disorder. Anovulation in women with PCOS is characterized by arrested follicle growth at the early antral stage, when AMH secretion from follicular granulosa cells is highest. Higher AMH concentrations consequently inhibit the recruitment of further primordial follicles, possibly representing more efficient use of the primordial ovarian pool^20^. This mechanism could possibly explain the consistent association we find between PCOS-susceptibility alleles and higher serum AMH concentrations, and might be a further mechanism towards slower ovarian ageing. Alternatively, higher AMH concentrations could indicate a larger ovarian primordial follicle pool size^4^. Such evolutionary debates are not just interesting arguments, but may be eventually informative to clinical practice. The anticipated persistence of reproductive lifespan may inform the use of artificial reproductive therapies or long-term lifestyle intervention strategies in women with PCOS.

Progress in identifying PCOS-susceptibility variants has been slow compared with other complex diseases, in part due to the relatively small collections of cases^10^. We demonstrate here, as previously reported for other traits^41^, that online self-reports of disease status is a highly efficient study design to identify large numbers of disease cases, providing sufficient power to identify robust genetic signals for PCOS. This is evident by our confirmation of previously identified PCOS signals in Han Chinese, by the highly consistent validation of our novel loci in cases defined by stringent clinical criteria and by the lack of heterogeneity in variant effect sizes between these case groups. That said, it remains important to confirm any findings of self-reported case studies in clinically validated cases.

The range of biological mechanisms that we can currently test by Mendelian randomization is limited by available GWAS findings. In particular, future analyses are needed to investigate the roles of androgen production and activity once robust genetic markers for those traits are identified. Indeed, we anticipate that future genetic instruments will allow wider and deeper testing of causal biological pathways. Although such analyses cannot infer possible developmental stage-specific effects of these pathways, the findings should encourage experimental studies that target these pathways, both to confirm the causal inferences and also to inform effective intervention and preventive strategies.

In conclusion, this genetic study reveals new biological and evolutionary insights into the pathogenesis of PCOS, including a major role of EGFRs, a co-primary neuroendocrine pathogenesis and genetic mechanisms towards slower ovarian ageing. Furthermore, the causal inferences from our Mendelian randomization analyses should support future efforts to develop and test effective interventions, to reduce body weight and insulin resistance in the treatment and prevention of PCOS.

## Methods

### Discovery phase

Genome-wide SNP data were available on 5,184 women of White European ancestry with self-reported PCOS and 82,759 controls from the 23andMe study (see Supplementary Table 1 and Supplementary Note for details of the 23andMe study). Imputation was performed against the 1000 Genomes reference (March 2012 v3 release), yielding ∼9 M variants that passed imputation and minor allele frequency criteria. A logistic regression model adjusting for age- and study-specific principal components was performed assuming an additive allelic model including covariates for age and the top five principal components to account for residual population structure. Test statistics were further adjusted for the observed *λ*-value 1.041. 23andMe participants provided informed consent to take part in this research under a protocol approved by Ethical and Independent Review Services, an accredited institutional review board.

### Follow-up studies

From our discovery GWAS phase results, we selected for follow-up in additional studies: (a) all signals that showed at least suggestive associations (*P*<1 × 10^−6^) with PCOS (*N*=5 signals, where a signal is defined by the most significant SNP within a 1-Mb window; Table 1); (b) all possible signals for PCOS (*P*<1 × 10^−5^) located within 500 kb of signals previously reported in Han Chinese (*N*=3 signals; in/near *YAP1*, *THADA* and *DENND1A*); and (c) possible signals for PCOS near to biological candidate genes (*N*=2 signals; in/near *ERBB2/HER2* and *FSHB*). Follow-up was performed in three independent studies of clinically validated PCOS cases and control women: deCODE, Rotterdam and Boston (see Supplementary Table 1 and Supplementary Note for details and parameters of follow-up studies). Separate follow-up analyses were performed using PCOS case definitions either by Rotterdam 2003 criteria^1^ (1,875 cases from Rotterdam and deCODE) or by NIH criteria^2^ (861 cases from Boston and deCODE). Final association test statistics were produced from a combined meta-analysis of 7,229 cases and 181,645 controls across non-overlapping discovery and follow-up (2,045 cases and 98,886 controls) samples; as the two PCOS groups in deCODE include overlapping cases, only deCODE cases defined by NIH criteria were included in this combined meta-analysis. The follow-up studies were approved by local research ethics committees and all participants provided informed consent.

### Mendelian randomization analyses

Mendelian randomization is an analytical method to infer the unconfounded causal relationship between an exposure trait and an outcome, using genetic variants that are associated with the exposure trait and do not influence the outcome by other unrelated biological pathways (‘pleitropy')^21^. In both the 23andMe and Rotterdam studies, we approximated weighted multiple allele scores (single variables summarizing multiple genetic variants associated with a risk factor, as described by Dastani *et al*.^42^), to represent genetic instrumental variables for 15 traits (birth weight, BMI, height, age at menarche, age at menopause, dehydroepiandrosterone sulphate, SHBG, total cholesterol, high-density lipoprotein cholesterol, low-protein lipoprotein cholesterol, triglycerides, systolic and diastolic blood pressure, insulin resistance and insulin secretion) based on reported GWAS signals for those traits. Each score was calibrated to a 1-s.d. change in the exposure trait, using the published effect estimates of individual alleles on those traits in the replication stages of those GWAS reports (Supplementary Table 3). To account for the multiple traits tested, we set a corrected *P*-value threshold (0.05/15=0.0033) to indicate statistically significant associations. To test for pleiotropy, which can invalidate inferences from Mendelian randomization, we performed sensitivity analyses to examine the consistency in causal estimates derived from individual SNPs.

### Serum AMH concentrations

The cumulative influence of PCOS-associated variants on childhood serum AMH concentrations, a marker of ovarian primordial follicle pool size^4^, was estimated by analysis of data in 1,455 girls (aged 15 years) from the ALSPAC study^43^. Serum AMH concentrations (ng ml^−1^) were natural log transformed before analysis in an additive linear regression framework.

### Tests for positive selection

Allelic variants that increase the reproductive fitness of their carriers should become more prevalent in the population. The resulting genomic characteristics of strong recent positive selection include low haplotype diversity, high linkage disequilibrium and marked shifts in allele frequency between populations. However, there is often poor consistency between signals identified from available tests^38^,^39^. We therefore looked for evidence of selection at the ten PCOS loci in Table 1, using various strategies.

We investigated whether any of the lead SNPs overlapped with signals of positive selection identified in 1000 Genomes data using the composite of multiple signals test^44^. None of the lead PCOS SNPs lies in any of the 424 non-overlapping regions with evidence of positive selection, a total of 19 Mb of sequence (http://www.broadinstitute.org/mpg/cmsviewer/download/cms_localized_regions_062712.txt). Three of the ten signals lie within 1 Mb of one of these regions (a total of 726 Mb of sequence), which is not more than expected by chance (*P*=0.56 assuming an accessible genome length of 2.6 Gb).

We tested whether the lead PCOS SNPs are more differentiated across populations compared to with randomly chosen loci, using the test described by Berg and Coop^45^, and Omni chip data from phase 1 of the 1000 Genomes Project^46^ as a reference panel (ftp://ftp-trace.ncbi.nih.gov/1000genomes/ftp/phase1/analysis_results/supporting/omni_haplotypes/). As only two of the ten PCOS SNPs were genotyped by the Omni chip, we added the remaining eight SNPs from the sequence data. Using 10,000 bootstrap replicates of SNP frequency matched in 20 bins, we find no evidence of polygenic selection in European (*P*=0.38), Asian (*P*=0.37), or combined European and Asian (*P*=0.42) populations.

We also tested PCOS susceptibility variants with minor allele frequency >0.2 using the integrated haplotype score^38^, which measures the difference in haplotype homozygosity associated with the ancestral and derived alleles, and the derived intra-allelic nucleotide diversity test^38^, which measures the differences in nucleotide diversity associated with the ancestral and derived alleles. We find no significant test statistics (*P*<0.01).

### Pathway analyses

MAGENTA (https://www.broadinstitute.org/mpg/magenta/) was used to test for enrichment of genome-wide SNP associations with PCOS in pre-defined biological pathways (Gene Ontology, PANTHER, KEGG and Ingenuity) using the full discovery data set. MAGENTA implements a gene-set enrichment analysis-based approach, where each gene throughout the genome is mapped to a single index SNP with the lowest *P*-value within a 110-kb upstream and 40-kb downstream window. This *P*-value, representing a gene score, is then corrected for confounding factors such as gene size, SNP density and linkage disequilibrium (LD)-related properties in a regression model. Genes within the human leukocyte antigen region were excluded from analysis, owing to difficulties in accounting for gene density and LD patterns. Each gene is then ranked by its adjusted gene score. At a given significance threshold (95th or 75th percentiles of all gene scores), the observed number of gene scores in a given pathway, with a ranked score above the specified threshold percentile, is calculated. This observed statistic is then compared with 1,000,000 randomly permuted pathways of identical size. This generates an empirical gene-set enrichment analysis *P*-value for each pathway. In total, 2,529 pathways were tested for enrichment of multiple modest associations with PCOS. Significant pathways are indicated by a false discovery rate <0.05 in either model (95th or 75th percentiles).

## Additional information

**How to cite this article:** Day, F. R. *et al*. Causal mechanisms and balancing selection inferred from genetic associations with polycystic ovary syndrome. *Nat. Commun.* 6:8464 doi: 10.1038/ncomms9464 (2015).

## Supplementary Material

Supplementary Information Supplementary Figures 1-3, Supplementary Tables 1-2, Supplementary Notes 1-2 and Supplementary References

Supplementary Data 1 Variants used in Mendelian Randomisation analysis

## Figures and Tables

**Table 1:** Genetic variants associated with risk of PCOS.

**Region**	**Gene**	**SNP**	Alleles*	**Discovery (self-reported)**		**Follow-up (2003 Rotterdam criteria)**		**Follow-up (NIH criteria)**		**Combined**†	
				**Effect**	***P-*****values**	**Effect**	***P***-**values**	**Effect**	***P***-**values**	**Effect**	***P***-**values**
*Novel loci*											
2q34	*ERBB4*	rs1351592	G/C/0.17	1.16 (1.10–1.22)	3.0E−08	1.16 (1.06–1.27)	1.2E−03	1.34 (1.15–1.56)	1.6E−04	1.18 (1.13–1.23)	1.2E−12
11q22.1	*YAP1*‡	rs11225154	A/G/0.09	1.16 (1.08–1.24)	2.6E−05	1.37 (1.23–1.54)	5.6E−08	1.24 (1.02–1.51)	3.1E−02	1.22 (1.15–1.29)	7.6E−11
2p21	*THADA*§	rs7563201	G/A/0.54	1.11 (1.07–1.16)	1.1E−06	1.13 (1.05–1.22)	1.2E−03	1.19 (1.05–1.36)	6.2E−03	1.13 (1.09–1.17)	3.3E−10
11p14.1	*FSHB*	rs11031006	A/G/0.14	1.11 (1.05–1.18)	2.9E−04	1.25 (1.14–1.37)	4.4E−06	1.29 (1.11–1.52)	1.4E−03	1.16 (1.11–1.22)	1.3E−09
5q31.1	*RAD50*	rs13164856	T/C/0.73	1.13 (1.07–1.18)	8.8E−07	1.15 (1.06–1.25)	8.0E−04	1.15 (1.00–1.32)	5.4E−02	1.13 (1.09–1.18)	3.5E−09
12q21.2	*KRR1*	rs1275468	C/T/0.75	1.13 (1.08–1.19)	8.0E−07	1.12 (1.03–1.22)	1.1E−02	1.13 (0.98–1.31)	9.5E−02	1.13 (1.08–1.18)	1.9E−08
											
*Suggestive loci*											
12q13.2	*ERBB3*||	rs7312770	C/T/0.47	1.11 (1.07–1.16)	9.0E−07	1.04 (0.97–1.12)	2.6E−01	1.15 (1.02–1.30)	2.4E−02	1.1 (1.06–1.15)	2.1E−07
17q12	*ERBB2*	rs7218361	A/G/0.04	1.24 (1.12–1.37)	2.9E−05	1.17 (0.95–1.44)	1.5E−01	1.36 (1.00–1.85)	4.7E−02	1.25 (1.14–1.37)	9.6E−07
9q33.3	*DENND1A*¶	rs10760321	A/G/0.71	1.13 (1.07–1.19)	2.7E−06	1.07 (0.98–1.16)	1.5E−01	1.09 (0.94–1.26)	2.4E−01	1.11 (1.07–1.16)	1.4E−06
											
*Unconfirmed*											
8q23.3	*TRPS1*	rs7012056	T/C/0.98	1.99 (1.5–2.6)	2.3E−07	1.15 (0.8–1.65)	4.4E−01	0.88 (0.48–1.61)	6.8E−01	1.55 (1.24–1.94)	1.4E−04

PCOS, polycystic ovary syndrome; SNP, single-nucleotide polymorphism.

^*^Effect allele (risk increasing)/other allele/effect allele frequency.

^†^The two follow-up arms contain some overlapping cases (that is, some cases satisfy both clinical criteria); however, only unique cases were used in the combined meta-analysis.

^‡^Reported locus (*R*^2^ 0.47).

^§^Reported locus (*R*^2^ 0.08).

^||^Reported locus (*R*^2^ 0.40).

^¶^Reported locus (*R*^2^ 0.02).

**Table 2:** PCOS associations in white Europeans for PCOS variants previously reported in Han Chinese.

**Region**	**Genes**	**SNP**	**Risk allele**	**Chinese**			White Europeans*		
				**RAF**	***P-*****values**	**OR**	**RAF**	**OR**	***P-*****values**
11q22.1	*YAP1*	rs1894116	G	0.19	1.0E−22	1.27	0.09	1.14 (1.06–1.22)	3.2E−04
12q13.2	*RAB5B/SUOX*	rs705702	G	0.25	9.0E−26	1.27	0.32	1.08 (1.03–1.12)	9.4E−04
2p21	*THADA*	rs13429458	A	0.91	4.0E−13	1.49	0.88	1.10 (1.03–1.17)	4.8E−03
16q12.1	*TOX3*	rs4784165	G	0.33	4.0E−11	1.15	0.26	1.07 (1.02–1.12)	6.6E−03
2p16.3	*FSHR*	rs2268361	C	0.50	1.0E−12	1.15	0.36	1.05 (1.01–1.1)	1.6E−02
12q14.3	*HMGA2*	rs2272046	A	0.91	2.0E−21	1.43	0.98	1.18 (1.02–1.37)	2.1E−02
19p13.2	*INSR*	rs2059807	G	0.30	1.0E−08	1.14	0.61	0.97 (0.93–1.01)	1.0E−01
2p16.3	*LHCGR*	rs13405728	A	0.76	4.0E−09	1.35	0.95	1.07 (0.97–1.17)	1.6E−01
20q13.2	*SUMO1P1*	rs6022786	A	0.34	2.0E−09	1.13	0.43	1.01 (0.97–1.06)	5.2E−01
9q33.3	*DENND1A*	rs2479106	G	0.22	5.0E−10	1.35	0.30	1.01 (0.97–1.06)	6.2E−01
9q22.32	*C9orf3*	rs3802457	G	0.90	5.0E−14	1.3	0.98	1.03 (0.9–1.18)	6.2E−01

OR, odds ratio; PCOS, polycystic ovary syndrome; RAF, risk allele frequency; SNP, single-nucleotide polymorphism.

^*^Estimates are from our discovery samples.

**Table 3:** Mendelian randomization analyses for PCOS risk.

**Trait**	**23andMe study**		**Rotterdam study**		**Combined**		
	Effect*	***P-*****values**	Effect*	***P-*****values**	Effect*	***P-*****values**†	***P***_**heterogeneity**_
BMI	2.05 (1.63–2.57)	5.6E−10	1.20 (0.71–2.03)	0.49	1.90 (1.55–2.34)	2.5E−09	0.07
Age at menopause	1.60 (1.35–1.91)	1.3E−07	1.57 (1.02–2.43)	0.04	1.60 (1.35–1.91)	1.5E−08	0.94
SHBG	0.86 (0.79–0.95)	0.002	0.81 (0.64–1.03)	0.08	0.86 (0.78–0.93)	5.4E−04	0.62
Insulin resistance	1.11 (1.04–1.19)	0.003	1.16 (0.99–1.36)	0.06	1.11 (1.05–1.19)	5.6E−04	0.59
DHEAS	1.11 (0.99–1.23)	0.06	—	—	—	—	—
HDL cholesterol	0.37 (0.13–1.11)	0.08	—	—	—	—	—
Insulin secretion	*Higher risk*	0.19	—	—	—	—	—
Birth weight	*Higher risk*	0.22	—	—	—	—	—
Age at menarche‡	0.91 (0.79–1.06)	0.23	—	—	—	—	—
Diastolic BP	1.01 (0.99–1.03)	0.24	—	—	—	—	—
LDL cholesterol	1.04 (0.94–1.16)	0.43	—	—	—	—	—
Adult height	*Lower risk*	0.51	—	—	—	—	—
Triglycerides	1.03 (0.90–1.18)	0.65	—	—	—	—	—
Systolic BP	1.05 (0.82–1.34)	0.68	—	—	—	—	—
Total cholesterol	0.98 (0.88–1.09)	0.71	—	—	—	—	—

BMI, body mass index; BP, blood pressure; DHEAS, dehydroepiandrosterone sulphate; HDL, high-density lipoprotein; LDL, low-protein lipoprotein; PCOS, polycystic ovary syndrome; SHBG, sex hormone binding globulin.

^*^Effect estimates are odds ratios for PCOS per 1 s.d. increase (based on s.d. from the genome-wide studies, approximated in the case of SHBG and DHEAS, as the discovery analysis used natural log units) in the candidate trait. For some traits, insufficient reported data were available to calculate an effect estimate, and in these cases only the direction of effect on PCOS risk is stated.

^†^Associations are displayed that passed the multiple test corrected *P*-value threshold (0.05/15=0.0033).

^‡^Any SNPs reported at genome-wide significance for adult BMI were omitted from this score.

## References

[b1] Rotterdam ESHRE/ASRM-Sponsored PCOS Consensus Workshop Group. Revised 2003 consensus on diagnostic criteria and long-term health risks related to polycystic ovary syndrome (PCOS). Hum. Reprod. 19, 41–47 (2004). 14688154 10.1093/humrep/deh098

[b2] Zawadzki J. & Dunaif A. in Polycystic Ovary Syndrome eds Dunaif A. G. J., Haseltine F. 377–384Blackwell Scientific (1992).

[b3] Amsterdam ESHRE/ASRM-Sponsored 3rd PCOS Consensus Workshop Group. Consensus on women's health aspects of polycystic ovary syndrome (PCOS). Hum. Reprod. 27, 14–24 (2012).22147920

[b4] Corbett S. & Morin-Papunen L. The polycystic ovary syndrome and recent human evolution. Mol. Cell. Endocrinol. 373, 39–50 (2013). 23352610 10.1016/j.mce.2013.01.001

[b5] Domecq J. P. . Lifestyle modification programs in polycystic ovary syndrome: systematic review and meta-analysis. J. Clin. Endocrinol. Metab. 98, 4655–4663 (2013). 24092832 10.1210/jc.2013-2385

[b6] Moran L. J., Hutchison S. K., Norman R. J. & Teede H. J. Lifestyle changes in women with polycystic ovary syndrome. Cochrane Database Syst. Rev. CD007506 (2011). 10.1002/14651858.CD007506.pub2 21328294

[b7] Dunaif A. Insulin resistance and the polycystic ovary syndrome: mechanism and implications for pathogenesis. Endocr. Rev. 18, 774–800 (1997). 9408743 10.1210/edrv.18.6.0318

[b8] Wild R. A. . Assessment of cardiovascular risk and prevention of cardiovascular disease in women with the polycystic ovary syndrome: a consensus statement by the Androgen Excess and Polycystic Ovary Syndrome (AE-PCOS) Society. J. Clin. Endocrinol. Metab. 95, 2038–2049 (2010). 20375205 10.1210/jc.2009-2724

[b9] Legro R. S. . Diagnosis and treatment of polycystic ovary syndrome: an Endocrine Society clinical practice guideline. J. Clin. Endocrinol. Metab. 98, 4565–4592 (2013). 24151290 10.1210/jc.2013-2350 PMC5399492

[b10] Barber T. M. & Franks S. Genetics of polycystic ovary syndrome. Front. Horm. Res. 40, 28–39 (2013). 24002403 10.1159/000341682

[b11] Chen Z. J. . Genome-wide association study identifies susceptibility loci for polycystic ovary syndrome on chromosome 2p16.3, 2p21 and 9q33.3. Nat. Genet. 43, 55–59 (2011). 21151128 10.1038/ng.732

[b12] Shi Y. . Genome-wide association study identifies eight new risk loci for polycystic ovary syndrome. Nat. Genet. 44, 1020–1025 (2012). 22885925 10.1038/ng.2384

[b13] Goodarzi M. O. . Replication of association of DENND1A and THADA variants with polycystic ovary syndrome in European cohorts. J. Med. Genet. 49, 90–95 (2012). 22180642 10.1136/jmedgenet-2011-100427 PMC3536488

[b14] Welt C. K. . Variants in DENND1A are associated with polycystic ovary syndrome in women of European ancestry. J. Clin. Endocrinol. Metab. 97, E1342–E1347 (2012). 22547425 10.1210/jc.2011-3478 PMC3387396

[b15] Mutharasan P. . Evidence for chromosome 2p16.3 polycystic ovary syndrome susceptibility locus in affected women of European ancestry. J. Clin. Endocrinol. Metab. 98, E185–E190 (2013). 23118426 10.1210/jc.2012-2471 PMC3537106

[b16] Louwers Y. V., Stolk L., Uitterlinden A. G. & Laven J. S. Cross-ethnic meta-analysis of genetic variants for polycystic ovary syndrome. J. Clin. Endocrinol. Metab. 98, E2006–E2012 (2013). 24106282 10.1210/jc.2013-2495

[b17] Saxena R. . Han Chinese polycystic ovary syndrome risk variants in women of European ancestry: relationship to FSH levels and glucose tolerance. Hum. Reprod. 30, 1454–1459 (2015). 25904635 10.1093/humrep/dev085 PMC4498224

[b18] Abbott D. H., Dumesic D. A. & Franks S. Developmental origin of polycystic ovary syndrome - a hypothesis. J. Endocrinol. 174, 1–5 (2002). 12098657 10.1677/joe.0.1740001

[b19] Escobar-Morreale H. F., Luque-Ramirez M. & San Millan J. L. The molecular-genetic basis of functional hyperandrogenism and the polycystic ovary syndrome. Endocr. Rev. 26, 251–282 (2005). 15561799 10.1210/er.2004-0004

[b20] Mulders A. G. . Changes in anti-Mullerian hormone serum concentrations over time suggest delayed ovarian ageing in normogonadotrophic anovulatory infertility. Hum. Reprod. 19, 2036–2042 (2004). 15217995 10.1093/humrep/deh373

[b21] Burgess S., Butterworth A., Malarstig A. & Thompson S. G. Use of Mendelian randomisation to assess potential benefit of clinical intervention. BMJ 345, e7325 (2012). 23131671 10.1136/bmj.e7325

[b22] Stolk L. . Meta-analyses identify 13 loci associated with age at menopause and highlight DNA repair and immune pathways. Nat. Genet. 44, 260–268 (2012). 22267201 10.1038/ng.1051 PMC3288642

[b23] Tang T., Lord J. M., Norman R. J., Yasmin E. & Balen A. H. Insulin-sensitising drugs (metformin, rosiglitazone, pioglitazone, D-chiro-inositol) for women with polycystic ovary syndrome, oligo amenorrhoea and subfertility. Cochrane Database Syst. Rev. 5, CD003053 (2012). 22592687 10.1002/14651858.CD003053.pub5

[b24] Moran L. J. . Dietary composition in the treatment of polycystic ovary syndrome: a systematic review to inform evidence-based guidelines. J. Acad. Nutr. Diet. 113, 520–545 (2013). 23420000 10.1016/j.jand.2012.11.018

[b25] Scott R. A. . Common genetic variants highlight the role of insulin resistance and body fat distribution in type 2 diabetes, independently of obesity. Diabetes 63, 4378–4387 (2014). 24947364 10.2337/db14-0319 PMC4241116

[b26] Ding E. L. . Sex hormone-binding globulin and risk of type 2 diabetes in women and men. N. Engl. J. Med. 361, 1152–1163 (2009). 19657112 10.1056/NEJMoa0804381 PMC2774225

[b27] Daan N. M. . Cardiovascular and metabolic profiles amongst different polycystic ovary syndrome phenotypes: who is really at risk? Fertil. Steril. 102, 1444–1451 (2014). 25239303 10.1016/j.fertnstert.2014.08.001

[b28] Costello M., Shrestha B., Eden J., Sjoblom P. & Johnson N. Insulin-sensitising drugs versus the combined oral contraceptive pill for hirsutism, acne and risk of diabetes, cardiovascular disease, and endometrial cancer in polycystic ovary syndrome. Cochrane Database Syst. Rev. CD005552 (2007). 17253562 10.1002/14651858.CD005552.pub2

[b29] Ciardiello F. & Tortora G. EGFR antagonists in cancer treatment. N. Engl. J. Med. 358, 1160–1174 (2008). 18337605 10.1056/NEJMra0707704

[b30] Park J. Y. . EGF-like growth factors as mediators of LH action in the ovulatory follicle. Science 303, 682–684 (2004). 14726596 10.1126/science.1092463

[b31] Jamnongjit M., Gill A. & Hammes S. R. Epidermal growth factor receptor signaling is required for normal ovarian steroidogenesis and oocyte maturation. Proc. Natl Acad. Sci. USA 102, 16257–16262 (2005). 16260720 10.1073/pnas.0508521102 PMC1283479

[b32] Sheng Q. & Liu J. The therapeutic potential of targeting the EGFR family in epithelial ovarian cancer. Br. J. Cancer 104, 1241–1245 (2011). 21364581 10.1038/bjc.2011.62 PMC3078592

[b33] Davies S. . High incidence of ErbB3, ErbB4, and MET expression in ovarian cancer. Int. J. Gynecol. Pathol. 33, 402–410 (2014). 24901400 10.1097/PGP.0000000000000081 PMC4153698

[b34] Hurtado A. . Regulation of ERBB2 by oestrogen receptor-PAX2 determines response to tamoxifen. Nature 456, 663–666 (2008). 19005469 10.1038/nature07483 PMC2920208

[b35] Chen D. J. . Two follicle-stimulating hormone receptor polymorphisms and polycystic ovary syndrome risk: a meta-analysis. Eur. J. Obstet. Gynecol. Reprod. Biol. 182C, 27–32 (2014). 25218548 10.1016/j.ejogrb.2014.08.014

[b36] Mason H. & Franks S. Local control of ovarian steroidogenesis. Baillieres Clin. Obstet. Gynaecol. 11, 261–279 (1997). 9536211 10.1016/s0950-3552(97)80037-5

[b37] Burt Solorzano C. M. . Neuroendocrine dysfunction in polycystic ovary syndrome. Steroids 77, 332–337 (2012). 22172593 10.1016/j.steroids.2011.12.007 PMC3453528

[b38] Fagny M. . Exploring the occurrence of classic selective sweeps in humans using whole-genome sequencing data sets. Mol. Biol. Evol. 31, 1850–1868 (2014). 24694833 10.1093/molbev/msu118

[b39] Kemper K. E., Saxton S. J., Bolormaa S., Hayes B. J. & Goddard M. E. Selection for complex traits leaves little or no classic signatures of selection. BMC Genomics 15, 246 (2014). 24678841 10.1186/1471-2164-15-246 PMC3986643

[b40] Perry J. R. . DNA mismatch repair gene MSH6 implicated in determining age at natural menopause. Hum. Mol. Genet. 23, 2490–2497 (2014). 24357391 10.1093/hmg/ddt620 PMC3976329

[b41] Tung J. Y. . Efficient replication of over 180 genetic associations with self-reported medical data. PLoS One 6, e23473 (2011). 21858135 10.1371/journal.pone.0023473 PMC3157390

[b42] Dastani Z. . Novel loci for adiponectin levels and their influence on type 2 diabetes and metabolic traits: a multi-ethnic meta-analysis of 45,891 individuals. PLoS. Genet. 8, e1002607 (2012). 22479202 10.1371/journal.pgen.1002607 PMC3315470

[b43] Anderson E. L. . Anti-mullerian hormone is not associated with cardiometabolic risk factors in adolescent females. PLoS One 8, e64510 (2013). 23762215 10.1371/journal.pone.0064510 PMC3675909

[b44] Grossman S. R. . Identifying recent adaptations in large-scale genomic data. Cell 152, 703–713 (2013). 23415221 10.1016/j.cell.2013.01.035 PMC3674781

[b45] Berg J. J. & Coop G. A population genetic signal of polygenic adaptation. PLoS. Genet. 10, e1004412 (2014). 25102153 10.1371/journal.pgen.1004412 PMC4125079

[b46] 1000 Genomes Project Consortium. . An integrated map of genetic variation from 1,092 human genomes. Nature 491, 56–65 (2012). 23128226 10.1038/nature11632 PMC3498066

