# Supplementary material for: Causal mechanisms and balancing selection inferred from genetic associations with polycystic ovary syndrome
Source: Nat Commun. 2015 Sep 29;6:8464. doi: 10.1038/ncomms9464 (PMC4598835; doi:10.1038/ncomms9464)
Supplement: Supplementary Information — Supplementary Figures 1-3, Supplementary Tables 1-2, Supplementary Notes 1-2 and Supplementary References [file ncomms9464-s1.pdf]

SUPPLEMENTARY FIGURES

Supplementary Figure 1 | Regional association plots for genome-wide significant PCOS signals. Dots represents individual SNP association P-values (on the –log10 scale) in the 23andMe discovery GWAS.

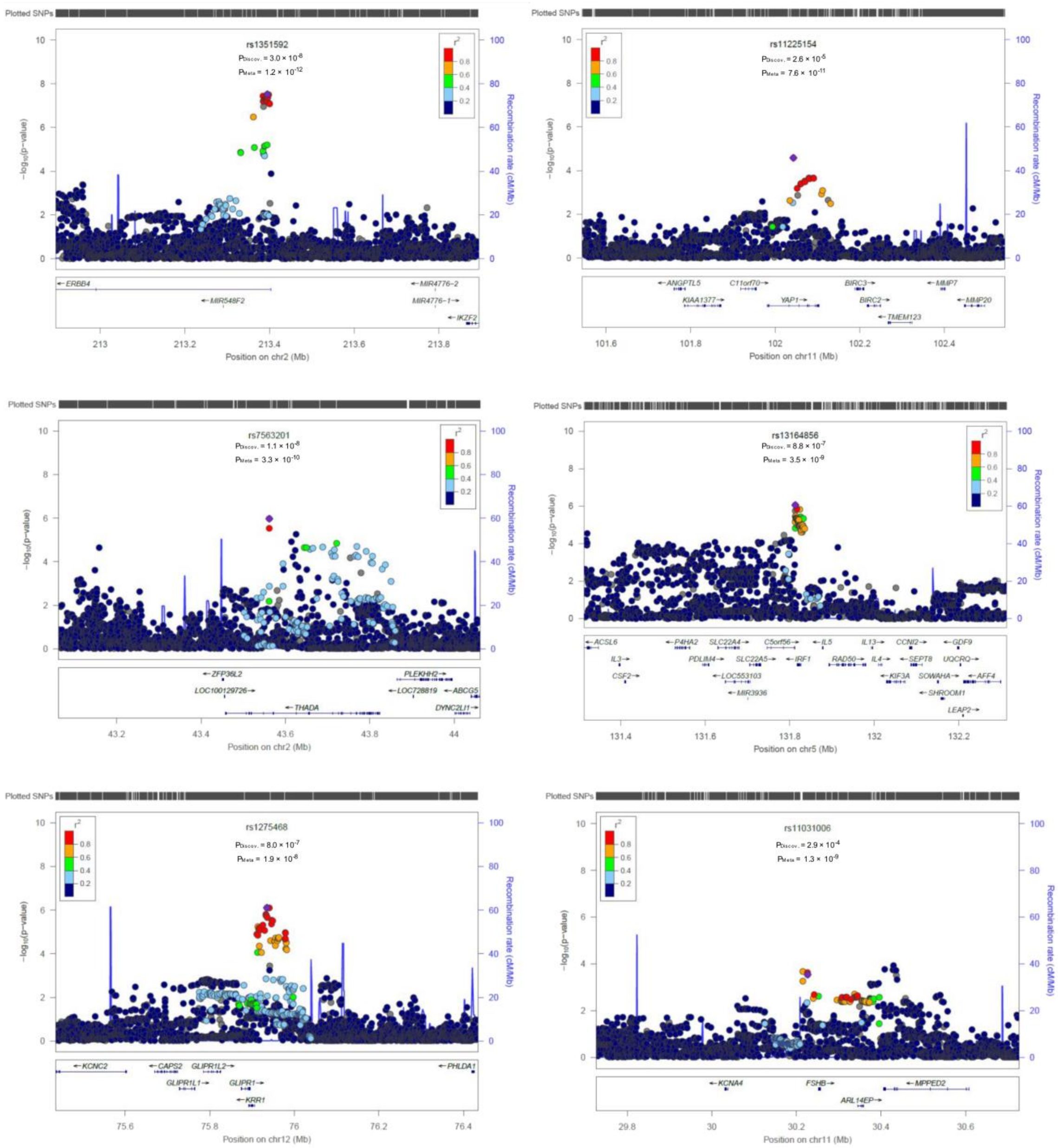

Supplementary Figure 2 |The impact of identified PCOS loci on serum AMH. Points represent individual effects of the six identified PCOS signals on serum AMH concentrations in ALSPAC study girls aged 15 years old.

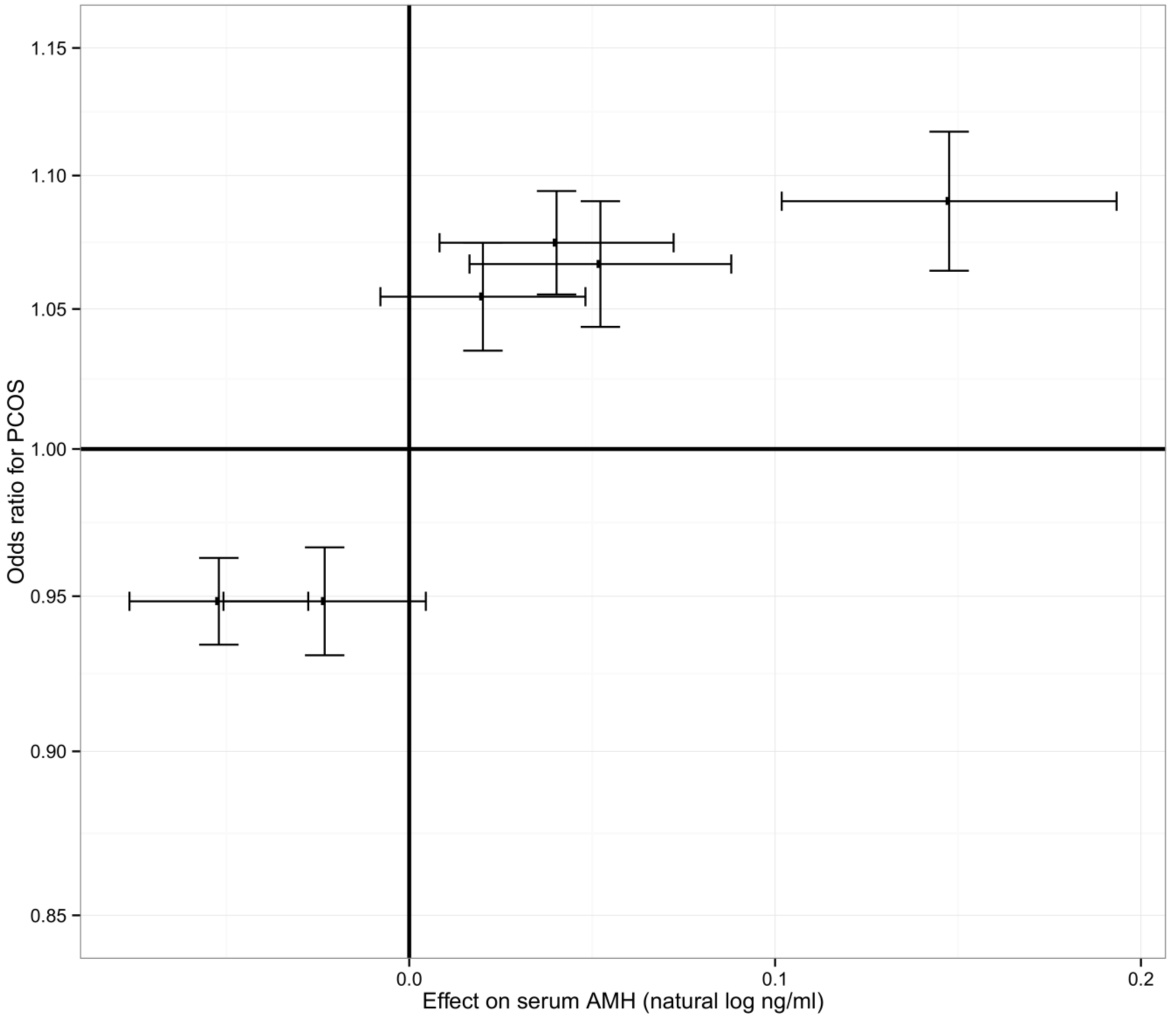

Supplementary Figure 3 | Funnel plots of the individual SNPs in each of the four significantly associated MR analyses. In each panel, the association with PCOS has been scaled to a one unit increase in the modelled trait – to allow comparison of the magnitude, and thus consistency in the effect estimates. The white area is centred around the averaged effect, with the dotted red line indicating the null effect. The white area enclosed is the 95% confidence interval, the wider dotted lines represent the 99% confidence interval. Across all four scores, only one SNP (rs17496332, near to *PRMT6* in the score for SHBG concentrations) was outside the 95% confidence intervals. In sensitivity tests omitting this SNP, the MR association between SHBG concentrations and PCOS remained significant: P=0.00011.

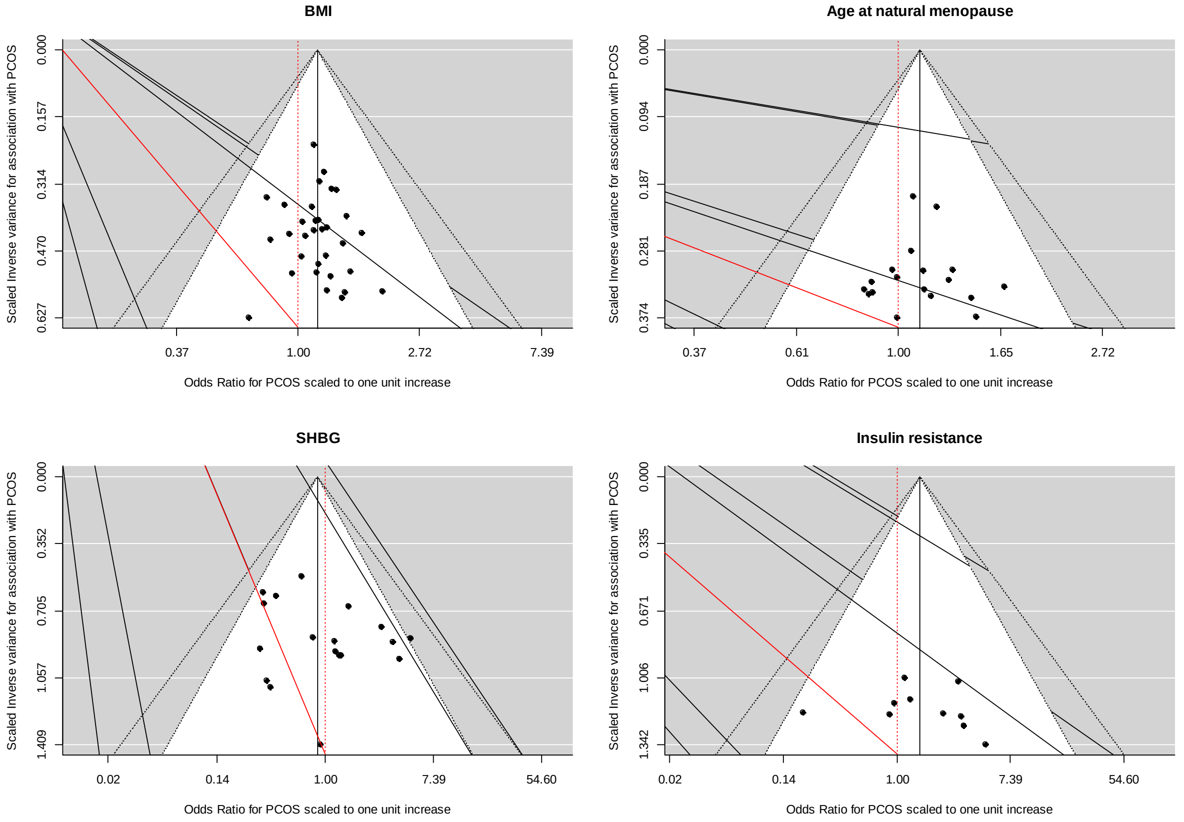

SUPPLEMENTARY TABLES

Supplementary Table 1 | Summarised characteristics of PCOS cases and controls in the discovery and follow-up studies

	23andMe		Rotterdam		Boston		Iceland		Iceland	
	Cases	Controls	Cases	Controls	Cases	Controls	Cases	Controls	Cases	Controls
**Number**	5184	82759	1184	5799	487	406	691	96182	374	92681
**Age (years)**	45.1 (13.6)	51.1 (15.7)	29.8 (6.3)	61.3 (8.4)	28.4 (6.7)	27.2 (6.5)	31.2 (8.6)	54.1 (18.6)	33.2 (9.5)	57.85 (18.2)
**BMI (kg/m2)**	29.2 (8.2)	26.1 (6.1)	26.4 (6.2)	27.6 (4.7)	30.8 (8.7)	23.8 (4.1)	29.6 (7.2)	26.3 (5.2)	30.4 (7.5)	26.6 (5.0)
**PCOS definition**	Self-report	-	2003 Rotterdam	-	NIH	-	2003 Rotterdam	-	NIH	-
**% menstrual irregularity**	-	-	86.6%	-	100%	-	56.1%	-	100%	-
**% hyperandrogenism (clinical or biochemical)**	-	-	43.4%	-	100%	-	98.0%	-	100%	-
**% polycystic ovarian morphology**	-	-	52.4%	-	90.9%	-	77.6%	-	58.6%	-

Means (SD) or % are displayed

Supplementary Table 2 | Heterogeneity in SNP-disease association estimates between discovery GWAS results in self-reported PCOS case studies versus studies using specified PCOS criteria.

Gene	SNP	Effect in Discovery GWAS	Effect in 2003 Rotterdam criteria studies	Effect in NIH criteria studies	Discovery versus 2003 Rotterdam criteria		Discovery versus NIH criteria	
					I^2	P-value	I^2	P-value
**Novel loci**								
ERBB4	rs1351592	1.16 [1.10-1.22]	1.16 [1.06-1.27]	1.34 [1.15-1.56]	0	0.9411	39.4	0.1912
YAP1	rs11225154	1.16 [1.08-1.24]	1.37 [1.23-1.54]	1.24 [1.02-1.51]	75.7	0.0163	0	0.8152
THADA	rs7563201	1.11 [1.07-1.16]	1.13 [1.05-1.22]	1.19 [1.05-1.36]	0	0.6172	0	0.5324
FSHB	rs11031006	1.11 [1.05-1.18]	1.25 [1.14-1.37]	1.29 [1.11-1.52]	70.2	0.0351	37.4	0.2023
RAD50	rs13164856	1.13 [1.07-1.18]	1.15 [1.06-1.25]	1.15 [1.00-1.32]	0	0.8785	0	0.9714
KRR1	rs1275468	1.13 [1.08-1.19]	1.12 [1.03-1.22]	1.13 [0.98-1.31]	0	0.9449	21.6	0.2794
**Suggestive loci**								
ERBB3	rs7312770	1.11 [1.07-1.16]	1.04 [0.97-1.12]	1.15 [1.02-1.30]	17.7	0.2965	0	0.7455
ERBB2	rs7218361	1.24 [1.12-1.37]	1.17 [0.95-1.44]	1.36 [1.00-1.85]	0	0.7603	0	0.7036
DENND1A	rs10760321	1.13 [1.07-1.19]	1.07 [0.98-1.16]	1.09 [0.94-1.26]	0	0.5387	0	0.8183
**Unconfirmed**								
TRPS1	rs7012056	1.99 [1.5-2.6]	1.15 [0.8-1.65]	0.88 [0.48-1.61]	64.7	0.0587	80.1	0.0065

SUPPLEMENTARY NOTES

SUPPLEMENTARY NOTE 1

GWAS discovery phase: the 23andMe Study

Participants were drawn from the customer base of 23andMe Inc, a consumer genetics company. All individuals provided informed consent and answered surveys online according to 23andMe's human subjects protocol, which was reviewed and approved by Independent Review Consulting, now part of Ethical & Independent Review Services, a private institutional review board (http://www.eandireview.com). 23andMe participants completed web-based questionnaires to record physician-diagnosed conditions. Polycystic ovary syndrome (PCOS) cases were defined as having stated "Yes" or reporting a diagnosis of PCOS to at least one of the following questions (under the following questionnaire categories):

- Female Fertility ("Have you ever been diagnosed with PCOS (polycystic ovary syndrome)?")
- Female Fertility ("What was your diagnosis? Please check all that apply." Answer = Polycystic ovarian syndrome (PCOS))
- Hair Loss in Men and Women ("Have you been diagnosed with any of the following? Please check all that apply." Answer = Polycystic ovary syndrome (PCOS))
- Research Snippets ("Have you ever been diagnosed with PCOS (Polycystic Ovary Syndrome)?")

Controls were defined as having said "No" or not reporting a diagnosis of PCOS to any of the questions above. Respondents who said they had PCOS in one question but said they didn't have it in another were excluded. Men were excluded from this analysis.

Genotyping was performed on one of three platforms, two based on the Illumina HumanHap550+ BeadChip and the third based on the Illumina Human OmniExpress+ BeadChip. Participants were restricted to a set of individuals who have >97% European ancestry, as determined by genetic analysis of local ancestry[1]. Briefly, the algorithm first partitions phased genomic data into short windows of about 100 SNPs. Within each window, a support vector machine (SVM) was used to classify individual haplotypes into one of 31 reference populations. The SVM classifications were then fed into a hidden Markov model (HMM) that accounts for switch errors and incorrect assignments, and gives probabilities for each reference population in each window. Finally, simulated admixed individuals were used to recalibrate the HMM probabilities so that the reported assignments are consistent with the simulated admixture proportions. The reference population data is derived from public datasets (the Human Genome Diversity Project, HapMap, and 1000 Genomes), as well as 23andMe customers who reported having four grandparents from the same country.

A maximal set of unrelated individuals was chosen for each analysis using a segmental identity-by-descent (IBD) estimation algorithm[2]. Individuals were defined as related if they shared more than 700 cM IBD, including regions where the two individuals share either one or both genomic segments identical-by-descent. This level of relatedness (roughly 20% of

the genome) corresponds approximately to the minimal expected sharing between first cousins in an outbred population.

Participant genotype data were imputed against the March 2012 “v3” release of 1000 Genomes reference haplotypes[3]. Data for each genotyping platform was phased and imputed separately. First Beagle[4] (version 3.3.1) was used to phase batches of 8000-9000 individuals across chromosomal segments of no more than 10,000 genotyped SNPs, with overlaps of 200 SNPs. SNPs with Hardy-Weinberg equilibrium $P<10^{-20}$, call rate < 95%, or with large allele frequency discrepancies compared to European 1000 Genomes reference data were excluded. Frequency discrepancies were identified by computing a 2x2 table of allele counts for European 1000 Genomes samples and 2000 randomly sampled 23andMe customers with European ancestry, and identifying SNPs with a chi squared $P<10^{-15}$. Each phased segment was imputed against all-ethnicity 1000 Genomes haplotypes (excluding monomorphic and singleton sites) using a high-performance version of Minimac[5], using 5 rounds and 200 states for parameter estimation.

Association test results were computed by logistic regression assuming additive allelic effects. For tests using imputed data, the imputed dosages were used rather than best-guess genotypes. Covariates for age and the top five principal components were used to account for residual population structure. Test statistics were further adjusted for the observed lambda value 1.041. The reported association test *P* values were computed using likelihood ratio tests.

SUPPLEMENTARY NOTE 2

<u>Follow-up studies:</u>

deCODE Study, Iceland

PCOS cases in Iceland were identified from the private practices of two gynecologists; from a study on obesity if a screening questionnaire indicated irregular menstrual cycles, hirsutism or acne; from a self-report of PCOS, advertisements, family members of PCOS probands or for other reasons.[6,7] All PCOS subjects in Iceland were examined by one of two research nurses trained by a Boston physician (CKW). Two sets of overlapping PCOS cases were identified as defined by NIH or 2003 Rotterdam criteria.[8,9]

All subjects arrived after a 12 hour fast and underwent a detailed history, physical examination including assessment of hirsutism, a transvaginal or transabdominal ultrasound and blood sampling. Late onset congenital adrenal hyperplasia was excluded with a follicular phase 17 OH progesterone <= 3.0 ng/mL (9.1 nmol/L). All subjects had normal thyroid function and prolactin levels and a follicular phase FSH level in the premenopausal range. Controls were selected from women in the deCODE Study who had no known features associated with PCOS such as hyperandrogenism, irregular menses or a history of polycystic ovary morphology or PCOS.[10] The study was approved by the Data Protection Commission of Iceland and the National Bioethics Committee of Iceland. All subjects gave their written informed consent.

Boston Study

PCOS cases in Boston, USA were recruited by advertisements or from reproductive endocrine or primary care outpatient clinics.[6,7] All subjects in Boston were examined by a reproductive endocrinologist or a physician assistant and fulfilled the NIH criteria for PCOS.[8]

All subjects arrived after a 12 hour fast and underwent a detailed history, physical examination including assessment of hirsutism, a transvaginal or transabdominal ultrasound and blood sampling. Late onset congenital adrenal hyperplasia was excluded with a follicular phase 17 OH progesterone <= 3.0 ng/mL (9.1 nmol/L). All subjects had normal thyroid function and prolactin levels and a follicular phase FSH level in the premenopausal range. Controls were recruited simultaneously and underwent the same research protocol. Subjects aged 18-37 years had menstrual cycle lengths between 25 and 35 days and subjects aged 37.5-45 years had menstrual cycle lengths between 21 and 35 days based on age-related follicular phase shortening. Controls had no self-reported or physical exam evidence of hirsutism and were not sisters of subjects fulfilling the Rotterdam criteria for the diagnosis of PCOS. The study was approved by the Institutional Review Board of the Massachusetts General Hospital. All subjects gave their written informed consent.

Rotterdam Study

PCOS cases in Rotterdam, the Netherlands were recruited from patients attending a large university medical center (the Erasmus MC University Medical Center).[11] Patients were diagnosed according to 2003 Rotterdam criteria.[9] Control women were derived from the Rotterdam study, a population-based prospective cohort study.[12] The study was approved by the medical ethics committee from the Erasmus MC University Medical Centre. All subjects provided fully written informed consent.

SUPPLEMENTARY REFERENCES

1. https://www.23andme.com/ancestry_composition_guide/ and https://customercare.23andme.com/hc/en-us/articles/202906830-Reference-populations-in-Ancestry-Composition .

2. Henn, B.M., Hon, L., Macpherson, J.M., Eriksson, N., Saxonov, S., Pe'er, I. & Mountain, J.L (2012). Cryptic distant relatives are common in both isolated and cosmopolitan genetic samples. *PLoS One* **7(4)**: e34267.

3. Durbin, R.M. et al. (2010) A map of human genome variation from population-scale sequencing. *Nature* **467**, 1061–1073.

4. Browning, S.R. & Browning, B.L. (2007) Rapid and accurate haplotype phasing and missing data inference for whole genome association studies using localized haplotype clustering. *Am. J. Hum. Genet.* **81**, 1084–1097.

5. Howie, B., Fuchsberger, C., Stephens, M., Marchini, J., & Abecasis, GR. (2012) Fast and accurate genotype imputation in genome-wide association studies through pre-phasing. *Nat. Genet.* **44**, 955-959.

6. Welt CK, Arason G, Gudmundsson JA, Adams J, Palsdóttir H, Gudlaugsdóttir G, Ingadóttir G, Crowley WF (2006). Defining constant versus variable phenotypic features of women with polycystic ovary syndrome using different ethnic groups and populations. *J Clin Endocrinol Metab*. **91**: 4361-4368.

7. Welt CK, Gudmundsson JA, Arason G, Adams J, Palsdottir H, Gudlaugsdottir G, Ingadottir G, Crowley WF (2006). Characterizing discrete subsets of polycystic ovary syndrome as defined by the Rotterdam criteria: the impact of weight on phenotype and metabolic features. *J Clin Endocrinol Metab*. **91**: 4842-4848.

8. Zawadzki JK, Dunaif A 1992 Diagnostic criteria for polycystic ovary syndrome: towards a rational approach. In: Dunaif A, Givens JR, Haseltine FP, Merriam GR, eds. Polycystic Ovary Syndrome. Boston: Blackwell Scientific; 377-384

9. ESHRE ASRM Sponsored PCOS Consensus Workshop Group (2004) Revised 2003 consensus on diagnostic criteria and long-term health risks related to polycystic ovary syndrome (PCOS). *Hum Reprod* **19**: 41–47.

10. Welt CK, Styrkarsdottir U, Ehrmann DA, Thorleifsson G, Arason G, Gudmundsson JA, Ober C, Rosenfield RL, Saxena R, Thorsteinsdottir U, Crowley WF, Stefansson K (2012). Variants in DENND1A are associated with polycystic ovary syndrome in women of European ancestry. *J Clin Endocrinol Metab*. **97**(7): E1342-7.

11. Louwers YV, Rayner NW, Herrera BM, Stolk L, Groves CJ, Barber TM, Uitterlinden AG, Franks S, Laven JS, McCarthy MI (2014). BMI-associated alleles do not constitute risk alleles for polycystic ovary syndrome independently of BMI: a case-control study. *PLoS One*. **9**(1): e87335.

12. Hofman A, Breteler MM, van Duijn CM, Janssen HL, Krestin GP, et al. (2009) The Rotterdam Study: 2010 objectives and design update. Eur J Epidemiol **24**: 553–572.
